# Constitutional *BRCA1* Methylation is associated with high level of tumoral *BRCA1* methylation and homologous recombination deficiency in triple-negative breast cancer

**DOI:** 10.1038/s41523-026-00906-3

**Published:** 2026-02-07

**Authors:** Justine Pasanisi, Constance Lamy, Lolita Lecompte, Sophie Vacher, Mathias Schwartz, Abderaouf Hamza, Sandrine M. Caputo, Sabrina Ibadioune, Laura Courtois, Frédérique Berger, Vincent Cockenpot, Sylvain Baulande, Jean-Yves Pierga, Celine Callens, Samia Melaabi, Chrystelle Colas, Eric Pasmant, Maud Kamal, Célia Dupain, Nicolas Servant, Christophe Le Tourneau, Ivan Bièche

**Affiliations:** 1 Department of Genetics, Institut Curie, Paris, France; 2 Department of Drug Development and Innovation (D3i), Institut Curie, Paris Saclay University, Paris, France; 3 Computational oncology, PSL Research University, Mines Paris Tech, INSERM 1331, Paris, France; 4 Paris Cité University, Paris, France; 5 Department of Biometry, Institut Curie, Saint-Cloud, France; 6 Department of Pathology, Institut Curie, Paris, France; 7 Genomics of Excellence (ICGex) Platform, Institut Curie, PSL Research University, Paris, France; 8 Department of Medical Oncology, Institut Curie, Paris, France

**Keywords:** Cancer, Genetics, Oncology

## Abstract

Tumoral *BRCA1* promoter methylation occurs frequently in triple-negative breast cancer (TNBC) and contributes to homologous recombination deficiency (HRD). While constitutional *BRCA1* methylation has been described, its relationship with tumoral methylation, genomic instability, and prognosis remains unclear. Paired tumor and blood samples from 136 TNBC patients (SCANDARE, NCT03017573) were analyzed for BRC*A1* methylation, genomic alterations, HRD and outcomes. Constitutional *BRCA1* methylation was detected in 20.6% of patients and tumoral methylation in 31.6%, including 11.5% with somatic-only methylation. In cases with constitutional *BRCA1* methylation, tumoral methylation levels increased markedly, with 89% of high-methylation tumors (≥50%) associated with a Loss of Heterozygoty. Tumors with *BRCA1* promoter methylation consistently exhibited high HRD (Homologous Recombination Deficiency) scores, comparable to those with pathogenic HRR (Homologous Recombination Repair) gene pathogenic variants (p < 0.001). Conversely, HRD was rare in tumors lacking both *BRCA1* methylation and HRR gene alterations. Prognostically, constitutional BRCA1 methylation tended to associate with improved survival, while somatic-only methylation showed a trend toward poorer outcomes (p = 0.06). Constitutional *BRCA1* methylation is associated with a high level of tumoral *BRCA1* promoter methylation and HRD in TNBC. These findings support integrating constitutional and tumoral *BRCA1* methylation into HRD assessment to improve patient stratification and precision treatment in TNBC.

## Introduction

Triple-negative breast cancer (TNBC) is a heterogeneous and aggressive subtype of breast cancer, defined by the absence of estrogen receptor (ER), progesterone receptor (PR), and HER2 overexpression^1^. Accounting for approximately 10–15% of breast cancers, TNBC is associated with poor prognosis and limited targeted treatment options^2–5^. In this context, epigenetic mechanisms have emerged as key players in TNBC biology.

Among them, tumoral *BRCA1* promoter methylation has attracted particular attention. Constitutional *BRCA1* promoter hypermethylation (*cBRCA1*) is observed in 5–8% of the general population^6–11^, and appears to be more frequent in TNBC patients, with a prevalence reaching approximately 20% in some cohorts^7,12^. Tumoral *BRCA1* methylation (*tBRCA1*) is a recurrent event in TNBC, associated with transcriptional silencing^13,14^ and impaired homologous recombination (HR)-mediated DNA repair^15^. However, the clinical significance of *BRCA1* promoter methylation is still debated, in part due to intratumoral heterogeneity^16^, and it is unclear whether constitutional and only somatic methylation (*sBRCA1*, detected in the tumor but absent in germline DNA) carry distinct clinical implications.

Homologous recombination deficiency (HRD) represents a key therapeutic vulnerability in TNBC, predicting sensitivity to platinum-based chemotherapy and PARP inhibitors^17,18^. Yet, response rates remain variable, underscoring the need for robust biomarkers to gain a better understanding of the HRD status beyond constitutional and tumoral HRR (Homologous Recombination Repair) gene mutations^19^.

In this study, we investigate the relationship between *BRCA1* promoter methylation, both constitutional and tumoral or only somatic, and HRD status in a large series of TNBC. By integrating *BRCA1* methylation, genomic, and clinical data, we aim to evaluate whether constitutional *BRCA1* methylation predisposes to tumoral *BRCA1* methylation and HRD in TNBC.

## Results

### *BRCA1* promoter methylation in blood and tumoral samples

**Fig. 1:**
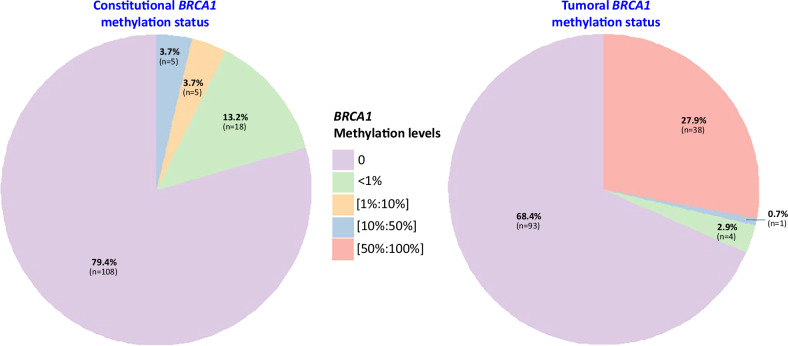
Distribution of constitutional and tumoral *BRCA1* promoter methylation levels in triple negative breast cancer patients. The methylation levels are divided into five categories: “0” (no methylation) in light purple, “<1%“ (low methylation) in light green, “[1%:10%]” (mild methylation) in yellow, “[10%:50%]” (moderate methylation) in light blue, and “[50%:100%]” (high methylation) in pink. The percentages (and number) of patients within each category are displayed on the chart.

We evaluated *BRCA1* promoter methylation in both constitutional and tumoral DNA from the 136 TNBC patients. As shown in Fig. 1, *BRCA1* promoter methylation was detected in 20.6% of constitutional samples and in 31.6% of tumoral samples, highlighting a high prevalence of *BRCA1* methylation in this breast cancer subtype. In constitutional DNAs, all *BRCA1* methylation levels were <50%, and no case exhibited high level of *BRCA1* methylation (≥50%). In contrast, 27.9% of tumoral samples showed high *BRCA1* promoter methylation levels (≥50%), of which 89% also exhibited loss of heterozygosity (LOH).

**Fig. 2:**
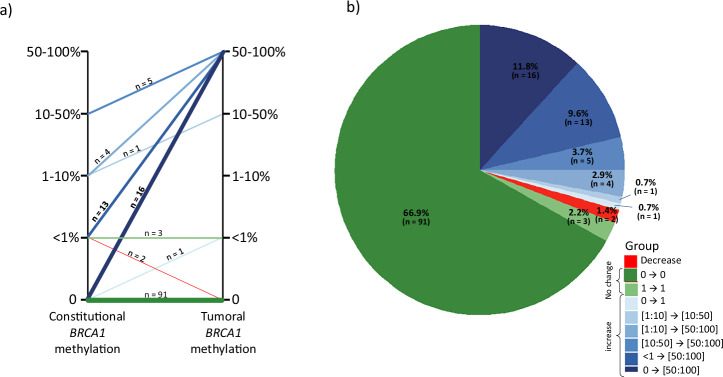
Dynamic of *BRCA1* promoter methylation level between matched constitutional and tumor samples. **a** Parallel plot and **b** pie chart illustrating differences in *BRCA1* methylation levels between constitutional and tumoral samples in TNBC patients. Red indicates a decrease in methylation levels, green represents stable methylation levels (including cases with no methylation), and blue shades indicate an increase in methylation levels (light blue for a small increase and dark blue for a substantial increase).

The individual evolution of *BRCA1* methylation levels from constitutional to tumoral DNA is shown in Fig. 2a, b. Among the patients with constitutional *BRCA1* methylation, almost all tumors also exhibited high tumoral *BRCA1* methylation levels, indicating a substantial increase in *BRCA1* methylation in the tumor context. Intermediate constitutional *BRCA1* methylation (1–50%) frequently progressed to higher levels in tumors, supporting a dynamic *BRCA1* methylation process during tumorigenesis. Moreover, 11.8% (16/136) of patients acquired high-level *BRCA1* methylation in tumor DNA despite no detectable *BRCA1* methylation in constitutional DNA. Decreases of *BRCA1* methylation in tumoral samples as compared to matched constitutional samples were rare (1.4%; *n* = 2).

### Relationship between *BRCA1* methylation status and HRD Status and HRR gene mutations

**Fig. 3:**
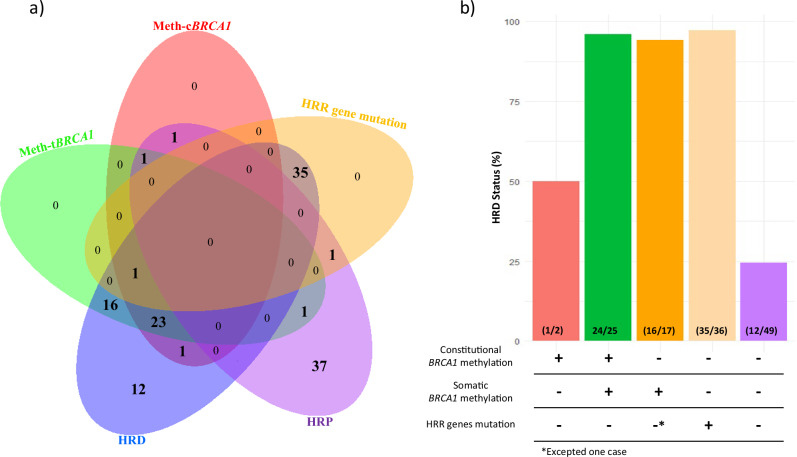
Association between *BRCA1* promoter methylation, HRR gene pathogenic variants and HRD status. **a** Venn diagram representing the overlap between *BRCA1* promoter methylation in constitutional (Meth-*cBRCA1*, red) and tumoral (Meth-*tBRCA1*, green) contexts, HRD (blue) and HRP (purple), status and the presence of mutations in HRR genes (yellow). Each region indicates the number of cases sharing one or more of these characteristics. **b** Proportion of HRD-positive tumors in each group defined by *BRCA1* methylation status (constitutional and/or tumoral) and HRR gene pathogenic variant status. The number of cases in each group is indicated within the bars. HRD: homologous recombination deficiency; HRP: homologous recombination proficiency**;** HRR: homologous recombination repair.

**Fig. 4:**
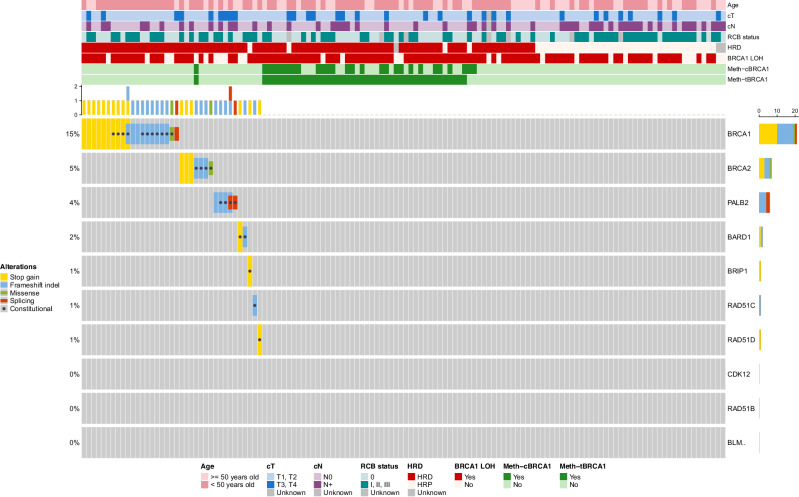
Epigenetic and genetic alterations in TNBC cohort. Oncoprint showing clinical characteristics (top rows), *BRCA1* methylation status (constitutional and tumoral), and HRD status, as well as alterations in 10 HRR genes (bottom panel) across the TNBC cohort. *BRCA1* methylation is indicated in dark green. HRD-positive tumors are shown in red. HRR gene pathogenic variants are shown according to type (stop gain, frameshift indel, missense, splicing) and constitutional origin (grey dot). HRD: homologous recombination deficiency; HRP: homologous recombination proficiency**;** RCB: residual cancer burden.

We explored the relationship between *BRCA1* methylation and HRD status and HRR gene mutations (Figs. 3 and 4; Table 1 and Supplementary Table 1). For this analysis, HRD scores were available for 133 out of the 136 TNBC cases, and information on mutations in HRR-related genes was available for 132 cases. 91 of the 133 (68%) tumors were HRD-positive. Among them, 41 (45%) had *BRCA1* methylation (constitutional and/or tumoral), while 35 (38.5%) carried pathogenic HRR gene variants (including *BARD1*, *BLM*, *BRCA1*, *BRCA2*, *BRIP1*, *CDK12*, *PALB2*, *RAD51B*, *RAD51C*, *RAD51D*). In 12 (13%) HRD cases, the underlying mechanism remained unknown (absence of *BRCA1* methylation and HRR gene pathogenic variants). Most homologous recombination proficient (HRP) tumors (37/41, 90%) lacked both *BRCA1* methylation and HRR gene pathogenic variant. Conversely, a total of three HRP tumors exhibited *BRCA1* methylation (two with c*BRCA1* methylation and one with s*BRCA1* methylation). All three patterns of *BRCA1* methylation, i.e. c*BRCA1* (constitutional), t*BRCA1* (tumoral) and s*BRCA1* (only somatic), were associated with HRD and the absence of HRR gene pathogenic variants (Table 1 and Supplementary Table 1). Only one tumor exhibited both *BRCA1* methylation and a HRR gene pathogenic variant (a constitutional *BRCA2* pathogenic variant; Fig. 4), suggesting a virtually complete mutual exclusivity between these two molecular mechanisms of homologous recombination deficiency.

**Table 1:** Association between *BRCA1* promoter methylation and HRD status or HRR gene mutations in TNBC patients

	Total population (%)	Non-methylated *cBRCA1*	Methylated *cBRCA1*	P value^a^	Non-methylated t*BRCA1*	Methylated t*BRCA1*	P value^a^	Non-Methylated s*BRCA1*	Methylated s*BRCA1*	P value^a^
**Total**	136 (100.0%)	108 (79.4%)	28 (20.6%)		93 (68.4%)	43 (31.6%)		119 (87.5%)	17 (12.5%)	
***HRD status***^***b***^				**0.0006**			**<0.0001**			**0.02**
HRP	42 (31.6%)	40 (37.7%)	2 (7.4%)		40 (44.0%)	2 (4.8%)		41 (35.3%)	1 (5.9%)	
HRD	91 (68.4%)	66 (62.3%)	25 (92.6%)		51 (56.0%)	40 (95.2%)		75 (64.7%)	16 (94.1%)	
***HRR constitutional mutations***^***c***^				**0.024**			**0.001**			**0.04**
Oui	24 (18.2%)	23 (22.1%)	1 (3.6%)		23 (25.8%)	1 (2.3%)		24 (20.9%)	0 (0.0%)	
Non	108 (81.8%)	81 (77.9%)	27 (96.4%)		66 (74.2%)	42 (97.7%)		91 (79.1%)	17 (100%)	
***HRR tumoral mutations***^***c***^				**0.001**			**<0.0001**			**0.006**
Oui	37 (28.0%)	36 (34.6%)	1 (3.6%)		36 (40.4%)	1 (2.3%)		37 (32.2%)	0 (0.0%)	
Non	95 (72.0%)	68 (65.4%)	27 (96.4%)		53 (59.6%)	42 (97.7%)		78 (67.8%)	17 (100%)	
***HRR somatic mutations***^***c***^				**0.03**			**0.004**			0.14
Oui	15 (11.4%)	15 (14.4%)	0 (0.0%)		15 (16.9%)	0 (0.0%)		15 (12.8%)	0 (0.0%)	
Non	117 (88.6%)	89 (85.6%)	28 (100%)		74 (83.1%)	43 (100%)		100 (87.2%)	17 (100%)	

^a^Chi-square test; ^b^Information available for 133 patients (3 non-contributory); ^c^Information available for 132 patients.

c*BRCA1* constitutional BRCA1, t*BRCA1* tumoral *BRCA1*, s*BRCA1* only somatic *BRCA1*, *HRD* Homologous recombination deficiency, *HRR* homologous recombination repair.

Bold values correspond to values statistically significant.

### Relationship between *BRCA1* methylation status and HRD scores

**Fig. 5:**
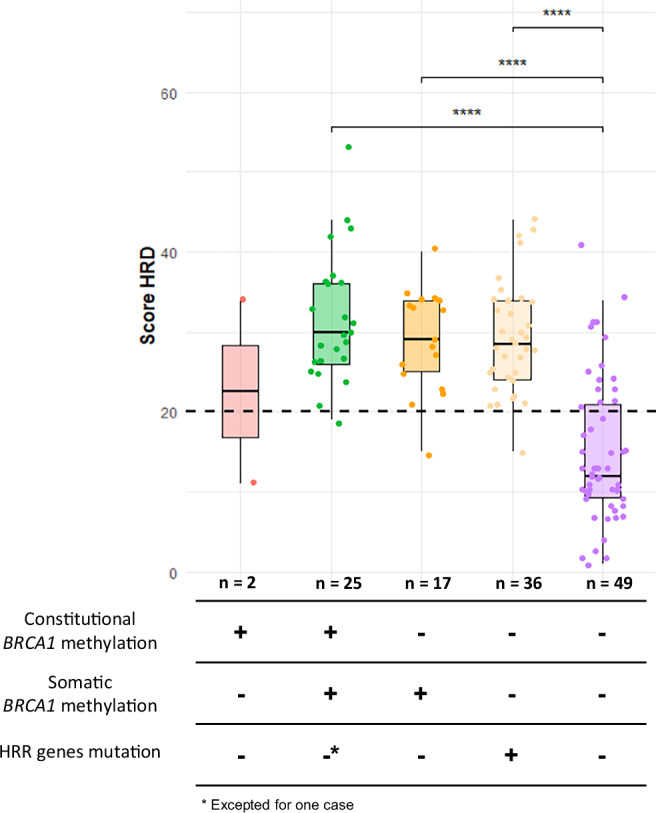
Distribution of HRD score across *BRCA1* methylation and HRR gene mutation groups. Tumors with somatic *BRCA1* methylation or HRR gene pathogenic variants show higher HRD scores, while most HRP tumors without *BRCA1* methylation or HRR alterations display lower HRD scores. *HRD* homologous recombination deficiency, *HRR* homologous recombination repair.

We next examined quantitatively the HRD scores across *BRCA1* methylation and HRR gene pathogenic variant groups (Fig. 5). Tumors with either *BRCA1* methylation (constitutional and/or tumoral) or pathogenic HRR gene variants displayed significantly higher HRD scores than tumors lacking both alterations, as largely described in the literature.

Notably, there was no significant difference in median HRD scores between tumors with combined constitutional and tumoral *BRCA1* methylation and those with only somatic *BRCA1* methylation (Fig. 5). Moreover, these two *BRCA1* methylated groups exhibited HRD scores comparable to those observed in HRR-mutated tumors, strongly suggesting that *BRCA1* promoter methylation induces a similar degree of homologous recombination deficiency as compared to HRR gene pathogenic variants. On the other hand, comparisons between cBRCA1 and sBRCA1-methylated tumors revealed no significant differences across the five non-HRR genes more frequently altered (frequency above 5%): PIK3CA, TP53, PTEN, RB1, and CDKN2A (Supplementary Table 2).

### Relationship between *BRCA1* methylation status and classical clinicopathological characteristics of the TNBC patients

To better characterize the clinical profiles linked to the three *BRCA1* methylation patterns, we examined their associations with patients’ clinicopathological characteristics (Supplementary Table 3). *tBRCA1* status a trend toward association with lymph node negativity (*non-adjusted p-value* = 0.03), and pathological response (RCB) status (*non-adjusted p-value* = 0.03). *sBRCA1* status showed a trend toward association with younger age at diagnosis (*non-adjusted p-value* = 0.03). However, these associations were not significant in adjusted *p*-value. Moreover, significant association was observed between *cBRCA1* status and any of the clinicopathological parameters studied.

### Prognostic impact of *BRCA1* methylation

**Fig. 6:**
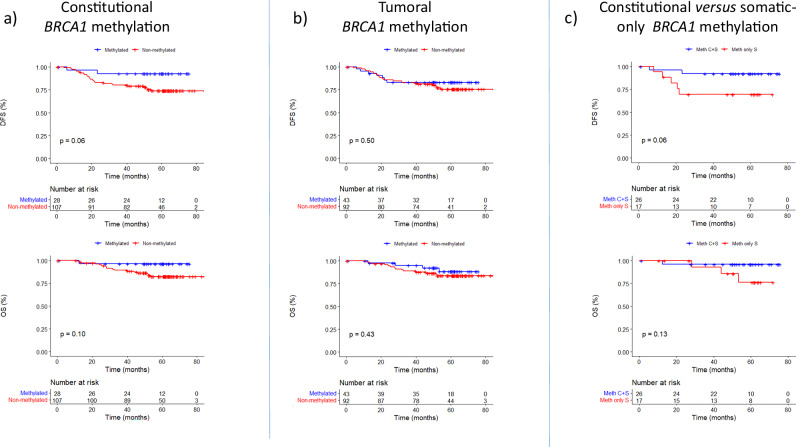
Kaplan–Meier curves for OS and DFS according to *BRCA1* methylation status in TNBC. **a** OS and DFS in patients stratified by c*BRCA1* status. **b** OS and DFS according to t*BRCA1* status. **c** OS and DFS comparing methylated *cBRCA1* and *sBRCA1* cBRCA1 constitutional *BRCA1* promoter, tBRCA1 tumoral *BRCA1* promoter methylation, sBRCA1 only somatic *BRCA1* promoter tBRCA1, OS overall survival, DFS disease free survival.

Follow-up data were available for 135 of the 136 TNBC patients to assess the prognostic impact of *BRCA1* methylation status. Survival analyses revealed no significant differences in overall survival (OS) or disease-free survival (DFS) between *BRCA1* methylated and unmethylated groups (Fig. 6a, b). Moreover, no significant association was observed between *BRCA1* methylation status and DFS, although a non-statistically significant trend toward improved DFS was noted in *cBRCA1* patients compared to those without constitutional *BRCA1* methylation (log-rank test, *p*-value = 0.06) (Fig. 6a). When comparing methylation patterns, *cBRCA1* patients showed a non-statistically significant trend toward better DFS than *sBRCA1* patients (*p*-value = 0.06) (Fig. 6c).

Finally, we explored the prognostic relevance of HRD status in our cohort. Follow-up data were available for 132 out of the 133 TNBC patients with known HRD status. HRD tumors were associated with better DFS compared to HRP tumors (*p*-value = 0.046) and showed a trend but non-significant towards better OS (*p* = 0.06) (Supplementary Fig. 1). These findings suggest that HRD status carries prognostic value in TNBC. Moreover, stratification according to adjuvant chemotherapy did not significantly impact OS (*p* = 0.18) or DFS (*p* = 0.17), suggesting that treatment heterogeneity is unlikely to account for the observed survival differences according to HRD status (Table 2 and Supplementary Fig. 2).

**Table 2:** Population characteristics and univariate overall survival analysis (N = 136)

Characteristic	N = 136^1^	N	HR^2^	95% CI^2^	p-value^3^
**Age at inclusion (years)**		136			NS
≤ 50 years	87 (64%)		—	—	
> 50 years	49 (36%)		0.67	0.24, 1.84	
**cT (Clinical TNM)**		136			0.02
T1-T2	104 (76%)		—	—	
T3-T4	32 (24%)		3.01	1.25, 7.24	
**cN (Clinical TNM)**		136			0.004
N0	80 (59%)		—	—	
N1 – N2 – N3	56 (41%)		3.82	1.47, 9.96	
**Histology**		136			NS
Ductal	130 (96%)		—	—	
Other	6 (4.4%)		1.22	0.16, 9.17	
**Elston-Ellis grade**		131			NS
Grade II	38 (29%)		—	—	
Grade III	93 (71%)		1.28	0.46, 3.57	
NA	5				
**NAC treatment**		134			NS
No NAC	4 (3.0%)		—	—	
Taxanes - Anthracyclines	117 (87%)		0.08	0.01, 0.66	
Other	13 (9.7%)		0.14	0.01, 1.42	
NA	2				
**Adjuvant treatment**		132			NS
No adjuvant treatment	83 (63%)		—	—	
Capecitabine	44 (33%)		1.41	0.55, 3.59	
Other	5 (3.8%)		1.44	0.18, 11.3	
NA	4				
**RCB status**		129			0.008
0	59 (46%)		—	—	
I-II-III	70 (54%)		4.33	1.25, 15.0	
NA	7				

^1^n (%).

^2^HR = Hazard Ratio, CI = Confidence Interval.

^3^log-rank test (Overall survival analysis).

## Discussion

In this study, we investigated the contribution of *BRCA1* promoter methylation, both at the constitutional and somatic level, to homologous recombination deficiency (HRD) and its potential prognostic value in TNBC. Constitutional *BRCA1* promoter methylation was detected in approximately 20% of TNBC cases, consistent with previous reports in the literature. Although the difference did not reach statistical significance, we observed a trend toward better survival among constitutionally methylated cases, suggesting a possible favorable prognostic impact that deserves further exploration in larger cohorts.

When assessing tumor DNA, the frequency of *BRCA1* promoter methylation increased to 31.6%, which is consistent with previous studies^20,21^. Among patients with constitutional methylation, tumor samples exhibited a striking increase in *BRCA1* methylation levels—from basal levels around 1% in constitutional DNA to exceeding 50% in the majority of the tumoral DNA cases—suggesting a two-step inactivation process mainly associated with a LOH “second hit”. No difference in survival outcomes was observed between patients with tumors with and without *BRCA1* methylation in tumor DNA, even though *tBRCA1* status is strongly associated with HRD status, which is itself associated with a trend toward better outcomes. This could be explained by the subset of *sBRCA1* patients (only somatically methylated cases) among *tBRCA1* patients, for which we observed an unexpected trend toward worse survival when compared to *cBRCA1* patients. These findings suggest that constitutional and somatic *BRCA1* methylation events may have distinct biological and clinical implications.

Interestingly, *BRCA1* only somatic methylation was more frequent in patients aged 50 or younger, and we also noted a trend toward poorer outcomes. This observation aligns with previous data reporting a more aggressive disease course in early-onset TNBC^22,23^, and raises the possibility that somatic *BRCA1* methylation in this context may contribute to a distinct, more aggressive phenotype. These findings of clinical and molecular associations should therefore be interpreted as exploratory and hypothesis-generating rather than definitive. Further investigations are needed to clarify whether age-associated epigenetic alterations influence tumor behavior or treatment response.

The integration of HRD analysis provided crucial insights. Nearly all tumors (except 3 cases) with *BRCA1* promoter methylation —whether constitutional, somatic, or both— displayed an HRD phenotype. Similarly, all tumors harboring pathogenic variants in HRR genes exhibited HRD, except one. Most notably, HRD scores were comparable in magnitude across constitutionally methylated, only somatically methylated, and HRR-mutated tumors, both in terms of HRD positivity and median score values. This reinforces the functional equivalence of these alterations in disrupting homologous recombination repair and supports the inclusion of *BRCA1* promoter methylation status in HRD-based stratification strategies. Such an approach is consistent with findings from the PAOLA-1/ENGOT-ov25 trial in ovarian cancer, where non-BRCA HRR gene alterations predicted benefit from olaparib plus bevacizumab^24^, highlighting the clinical relevance of broader HRD definition.

As classically described in the literature, a subset of tumors displayed an HRD phenotype in the absence of detectable HRR alterations or BRCA1 methylation was observed in our TNBC cohort. This finding suggests that additional mechanisms may contribute to homologous recombination impairment, including epigenetic deregulation of other HRR-related genes^25^, large genomic rearrangements not captured by short-read sequencing approaches or deep intronic splice altering variants affecting HRR gene function. Although these mechanisms were not directly assessed in the present study, they highlight the need for complementary genomic and epigenomic approaches to fully characterize HRD drivers in TNBC. Finally, consistent with previous studies^21,26,27^, we found that HRD-positive tumors were associated with improved survival outcomes. These results confirm HRD as a favorable prognostic marker in TNBC and suggest that epigenetic silencing of *BRCA1* contributes meaningfully to this phenotype, alongside genetic alterations. Altogether, our findings support the inclusion of *BRCA1* promoter methylation assessment—both constitutional and somatic—into the molecular landscape of TNBC, paving the way for an epigenetic dimension in precision oncology and expanding therapeutic opportunities beyond genetic testing alone.

## Methods

### Patients and sample collection

Tumor tissues and blood samples were obtained from TNBC patients diagnosed at Institut Curie (Paris and Saint-Cloud, France) between January 10, 2017, and September 2021, in the prospective biobanking SCANDARE study (NCT03017573). SCANDARE was approved by a national ethics committee (CPP Ile-de-France 3; Reference no. 3440) and by the National Agency for the Safety of Drugs and Health Products (ANSM; Reference: ID-RCB 2016-A01095-46). All patients provided frozen tissue of the initial biopsy specimen (*n* = 136) and paired whole blood samples, after patients’ consent had been obtained, in accordance with the Declaration of Helsinki. Moreover, 87% (117/134) of patients treated with neoadjuvant chemotherapy received a standard anthracycline- and taxane-based regimen. Comprehensive clinical, histological, and molecular data were collected for each case (Table 2 and Supplementary Table 1).

### DNA extraction

Frozen tissue fragments were incubated in a denaturing buffer containing proteinase K to digest proteins. RNA is then degraded by RNase treatment, and finally, DNA is extracted using buffered phenol and precipitated. DNA integrity and concentration were assessed using Nanodrop, Qubit, and TapeStation 4200 platforms.

From blood sample, germline DNA was extracted from whole blood using Quick gene DNA whole blood kit (FSVT fujifilm life science, Minato-ku, Tokyo, Japan).

### Genomic analyses

For, BRCA1 methylation analysis, semi-quantitative *BRCA1* promoter methylation evaluation was performed in DNA extracted from blood from all patients, using specific Methylation-Sensitive High-Resolution Melting (MS-HRM) EpiMelt assay from Methyl Detect ApS (Aalborg, Denmark, ref: MD-BRCA1) targeting six CpG sites of the core BRCA1 promoter (c.−133 to c.−96), after bisulphite conversion by the EZ DNA Methylation-Lightning kit (Zymo Research, Irvine, California, USA), as previously described (Schwartz et al. 2025). MS-HRM has been shown to be able to detect *BRCA1* promoter methylation with variant epiallele frequencies (VEFs) as low as 0.1%. Semi-quantitative results allow samples to be dispatched into four VEF groups: low-VEF ( < 1%), mild-VEF (1–10%) moderate-VEF (10–50%) and high-VEF ( ≥ 10%). This approach has been previously validated quantitatively by ddPCR, confirming its accuracy even at very low methylation levels.

For Whole Exome Sequencing (WES), Genomic DNA was sheared using the Covaris system. Library preparation was carried out using the Roche Kapa Hyper Exome Prep kit following the manufacturer’s protocol. Libraries were pooled in equimolar condition before being hybridized on dedicated KAPA Exome Enrichment Probes (KAPA_HyperExomeV2_hg38_capture_targets.bed). After selection using streptavidin beads and PCR amplification, enriched library pools were quantified using the KAPA library quantification kit (Roche, Basel, Switzerland). Sequencing was carried out on the NovaSeq 6000 instrument (Illumina, San Diego, California) (paired-end reads, 100 bases) on a S4 flow cell to obtain around 25 million clusters (50 million raw paired-end reads) per blood sample (30X target depth of coverage) and 75 million clusters (150 million raw paired-end reads) per tumor sample (100X target depth of coverage).

For Shallow Whole Genome Sequencing (shWGS), Kapa pre-capture pools were used for low-coverage whole genome sequencing of all tumor samples. Sequencing was carried out on a NovaSeq 6000 (paired-end reads, 100 bases) using a S1 flow cell to obtain around 20 million clusters (40 million raw paired-end reads) per sample.

### Bioinformatics analysis

Whole exome sequencing data were processed with the Institut Curie VEGAN pipeline (Servant et al. 2024) (v2.6.0 https://github.com/bioinfo-pf-curie/vegan). Briefly, reads were mapped to the human hg38 reference genome using BWA-MEM. Uniquely mapping reads intersecting the exome capture with a minimum mapping quality of 20 were kept for downstream analysis (SAMtools, BEDtools). Duplicate removal was performed using Picard MarkDuplicates tool.

Calling of somatic single-nucleotide variants (SNVs) and insertions/deletions (indels) was performed using MuTect2 (GATK v4.1.8.0). Somatic variants annotation was performed with SnpEff (v.5.1). Only coding variants satisfying the caller’s quality filters and the following criteria were reported: (i) tumor read depth ≥ 20, (ii) variant allele frequency ≥ 5 % for frozen samples, (iii) frequency in the general population equal or lower than 0.1% in gnomAD. Variants filtered by Mutect2 because they were annotated as ‘germline’ or ‘normal_artifact’ and present at a low frequency in the normal sample (<5%) were rescued. Loss of Heterozygosity (LOH) was determined using Facets (v0.6.1) on matched tumor-normal sample pairs.

Germline variant calling of SNVs and indels was performed using HaplotypeCaller (GATK v4.1.8.0). Germline variant annotation was performed with SnpEff (v.5.1). Only pathogenic variants for genes of interest (*BARD1, BLM, BRCA1, BRCA2, BRIP1, CDK12, PALB2, RAD51B, RAD51C, RAD51D*) were considered.

For the selection of driver mutations in the oncoprint, Inactivating variants were considered for TSG including (i) pathogenic missense variants known in the COSMIC, ICGC, or Cancer Hotspot databases, (ii) inframe indel variants known in the COSMIC, ICGC, or Cancer Hotspot databases, and (iii) truncating variants such as stop gains, frameshift indels, and variants disrupting splice sites. Oncoprint was generated using the R (v.4.4.1) package ComplexHeatmap (v.2.20.0).

HRD status was determined using the ShallowHRD v2 tool on shWGS data (Callens et al., 2023). The tool relies on a rule-based scoring system integrating large genomic alterations (LGA, segments of > 30 Mb) count, genomic complexity, and predefined binary markers (like ERBB2 amplification), with thresholds applied according to sample quality.

### Statistical analysis

The patient baseline characteristics are presented as numbers and percentages for the categorical variables, and as medians, ranges and means. Disease-free survival (DFS) is computed from the date of diagnosis to the date of loco-regional recurrence, distant metastasis, second cancer, or death, whichever comes first. Overall survival (OS) is defined as the time between the date of diagnosis and the date of death. Patients alive and free of events are censored at their date of last visit. DFS and OS are estimated using the Kaplan-Meier method and are presented graphically.

The statistical analyses were performed using R software (v.4.1.2) and the Survival R package (v.3.4-0) for the Kaplan-Meier method.

## Supplementary information

Supplementary Informations. PASANISI et al_revised

Supplementary Data 1

## Data Availability

SCANDARE TNBC shallow WGS and SCANDARE TNBC WES datasets are available under the accession numbers EGAD50000001849 and EGAD50000001661 (EGAC00001000670).

## Code availability

Source codes are given in https://github.com/bioinfo-pf-curie/SCANDARE/tree/main/Pasanisi_et_al.
