# Supplementary material for: Constitutional BRCA1 Methylation is associated with high level of tumoral BRCA1 methylation and homologous recombination deficiency in triple-negative breast cancer
Source: NPJ Breast Cancer. 2026 Feb 7;12:39. doi: 10.1038/s41523-026-00906-3 (PMC12996330; doi:10.1038/s41523-026-00906-3)
Supplement: Supplementary file 1 — Supplementary Informations. PASANISI et al_revised [file 41523_2026_906_MOESM1_ESM.pdf]

# HRD status

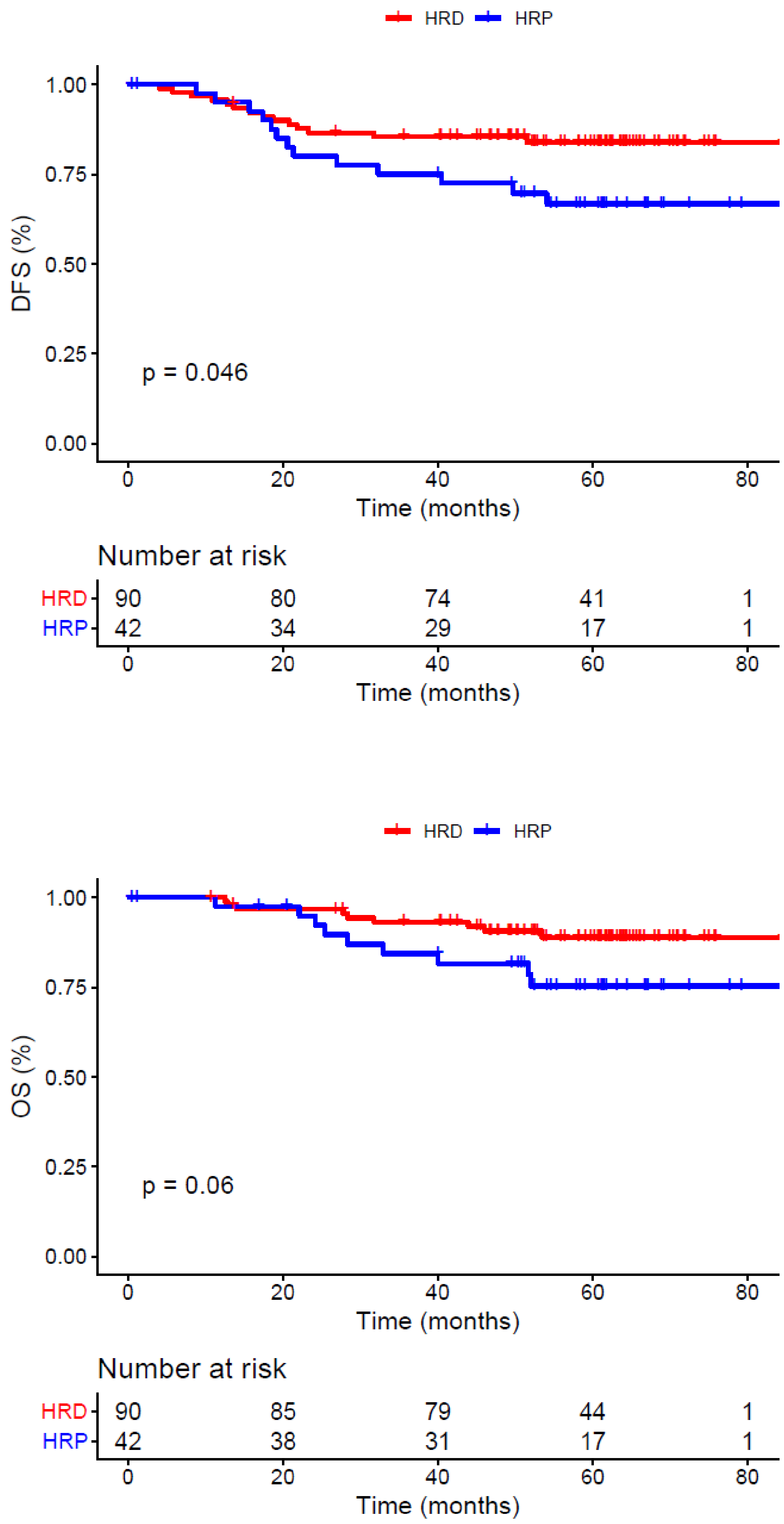


**Supplementary Figure 1. Kaplan–Meier curves for OS and DFS according to HRD status in TNBC**

OS and DFS in patients stratified by HRD status

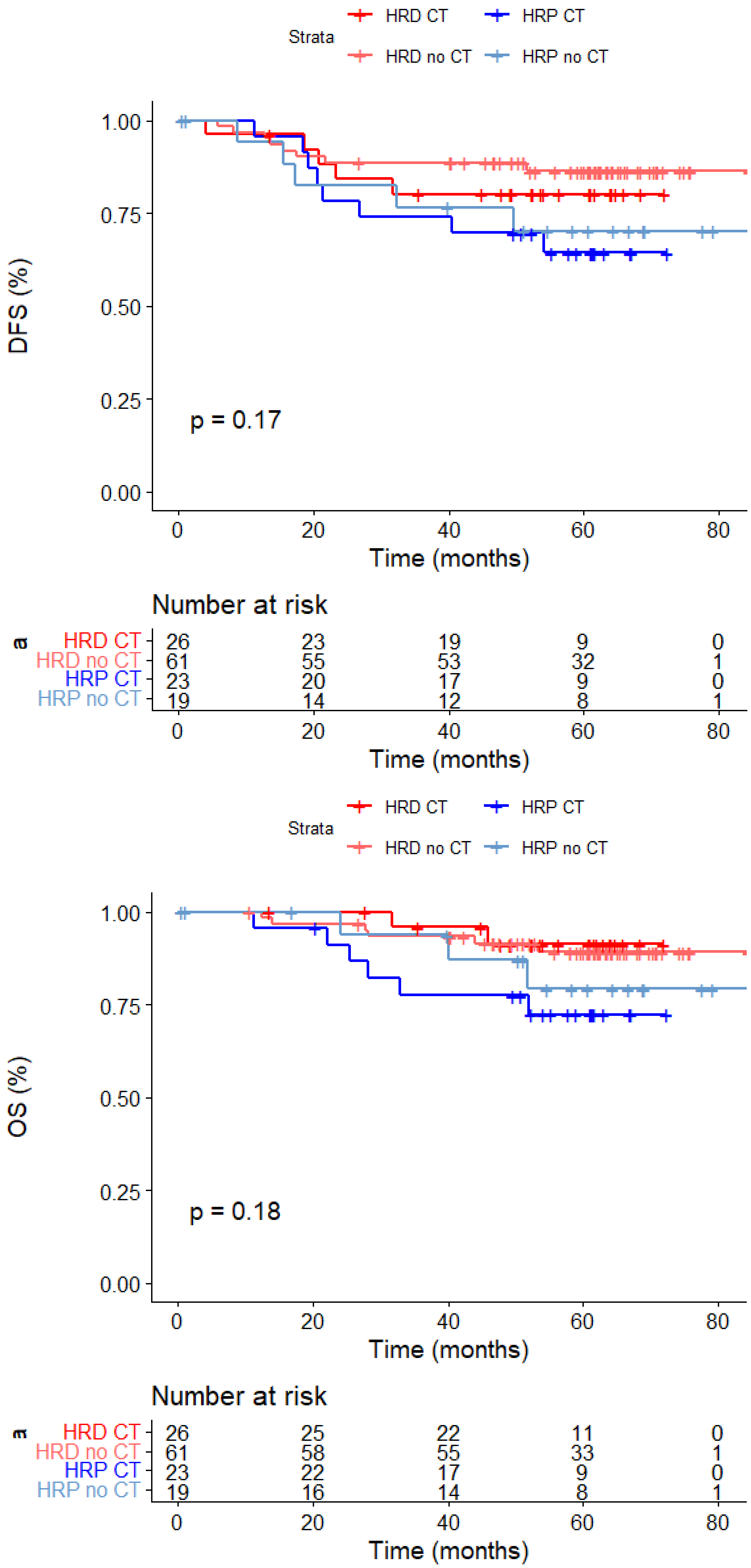


**Supplementary Figure 2. Kaplan–Meier curves for OS and DFS according to HRD status in TNBC treated or not with chemotherapy**

**Supplementary Table 2**. Frequency (%) of non HRR genes mutations among *cBRCA* and *sBRCA1* methylated patients

| | Constitutional *BRCA1* methylation | | Somatic *BRCA1* methylation | | |
|---|---|---|---|---|---|
| **Gene** | **Gene altered** | **Gene unaltered** | **Gene altered** | **Gene unaltered** | **Fisher's exact test *p*-value** |
| *PIK3CA* (mutations) | 2 (8%) | 23 (92%) | 0 (0%) | 17 (100%) | 0.5 |
| *RB1* (mutations or/and deletions) | 1 (4%) | 24 (96%) | 2 (6%) | 15 (94%) | 0.6 |
| *CDKN2A* (deletions) | 3 (12%) | 22 (88%) | 1 (6%) | 16 (94%) | 0.6 |
| *TP53* (mutations) | 23 (92%) | 2 (8%) | 15 (88%) | 2 (12%) | 1.0 |
| *PTEN (* mutations or/and deletions) | 4 (16%) | 21 (84%) | 2 (12%) | 15 (88%) | 1.0 |

Supplementary Table 3 : Characteristics of the 136 patients and relation to *BRCA1* methylation status

| | **Total population (%)** | **Non-methylated *cBRCA1*** | **Methylated *cBRCA1*** | **P value[a]** | **Non-methylated t*BRCA1*** | **Methylated t*BRCA1*** | **P value[a]** | **Non-Methylated s*BRCA1*** | **Methylated s*BRCA1*** | **P value[a]** |
|---|---|---|---|---|---|---|---|---|---|---|
| **Total** | 136 (100.0%) | 108 (79.4%) | 28 (20.6%) | | 93 (68.4%) | 43 (31.6%) | | 119 (87.5%) | 17 (12.5%) | |
| ***Age*** | | | | 0.08 | | | 0.85 | | | **0.03** |
| ≤ 50 years | 87 (64.0%) | 73 (67.6%) | 14 (50.0%) | | 59 (63.4%) | 28 (65.1%) | | 72 (60.5%) | 15 (88.2%) | |
| > 50 years | 49 (36.0%) | 35 (32.4%) | 14 (50.0%) | | 34 (36.6%) | 15 (34.9%) | | 47 (39.5%) | 2 (11.8%) | |
| ***cT (Clinical TNM)*** [b] | | | | 0.47 | | | 0.86 | | | 0.20 |
| T1 + T2 | 104 (77.0%) | 81 (75.7%) | 23 (82.1%) | | 71 (77.2%) | 33 (76.7%) | | 93 (78.8%) | 11 (64.7%) | |
| T3 + T4 | 31 (23.0%) | 26 (24.3%) | 5 (17.9%) | | 21 (22.8%) | 10 (23.3%) | | 25 (21.2%) | 6 (35.3%) | |
| ***cN (Clinical TNM)*** [b] | | | | 0.19 | | | **0.03** | | | 0.34 |
| Node-negative | 80 (59.3%) | 62 (57.9%) | 20 (71.4%) | | 50 (54.3%) | 32 (74.4%) | | 69 (58.5%) | 12 (70.6%) | |
| Node-positive | 55 (41.7%) | 45 (42.1%) | 8 (28.6%) | | 42 (45.7%) | 11 (25.6%) | | 49 (41.5%) | 5 (29.4%) | |
| ***Histology*** [b] | | | | 0.28 | | | 0.17 | | | 0.61 |
| Ductal | 130 (96.3%) | 104 (97.2%) | 26 (92.9%) | | 90 (97.8%) | 40 (93.0%) | | 114 (96.6%) | 16 (94.1%) | |
| Other | 5 (3.7%) | 3 (2.8%) | 2 (7.1%) | | 2 (2.2%) | 3 (7.0%) | | 4 (3.4%) | 1 (5.9%) | |
| ***Elston-Ellis grade*** [c] | | | | 0.40 | | | 0.71 | | | 0.23 |
| Grade I | 0 (0%) | | | | | | | | | |
| Grade II | 38 (28.8%) | 32 (30.5%) | 6 (22.2%) | | 25 (27.8%) | 13 (31.0%) | | 31 (27.0%) | 7 (41.2%) | |
| Grade III | 94 (71.2%) | 73 (69.5%) | 21 (77.8%) | | 65 (72.2%) | 29 (69.0%) | | 84 (73.0%) | 10 (58.8%) | |
| ***RCB status*** [d] | | | | 0.17 | | | **0.03** | | | 0.15 |
| 0 | 59 (45.7%) | 44 (42.7%) | 15 (57.7%) | | 35 (39.3%) | 24 (60.0%) | | 49 (43.4%) | 10 (62.5%) | |
| I + II + III | 70 (54.3%) | 59 (57.3%) | 11 (42.3%) | | 54 (60.7%) | 16 (40.0%) | | 64 (56.6%) | 6 (37.5%) | |

[a]Chi-square test;[b]Information available for 135 patients; [c]Information available for 132 patients; [d]Information available for 129 patients

c*BRCA1* = constitutional BRCA1; t*BRCA1* = tumoral *BRCA1* ; s*BRCA1* = somatic *BRCA1* ; RCB = Residual Cancer Burden
